# Visualizing exertional dyspnea in a post-COVID patient using electrical impedance tomography

**DOI:** 10.1007/s15010-023-02062-3

**Published:** 2023-06-15

**Authors:** Yvonne Gremme, Steffen Derlien, Katrin Katzer, Philipp A. Reuken, Andreas Stallmach, Jan-Christoph Lewejohann, Christina Lemhöfer

**Affiliations:** 1 Department of Internal Medicine IV (Gastroenterology, Hepatology and Infectious Diseases), Jena University Hospital/Friedrich-Schiller-Universität Jena, Jena, Germany; 2 Center for Sepsis Control and Care (CSCC), Jena University Hospital/Friedrich-Schiller-Universität Jena, Jena, Germany; 3 Department of Emergency Medicine, Jena University Hospital/Friedrich-Schiller-Universität Jena, Jena, Germany; 4 Institute of Physical and Rehabilitation Medicine, Jena University Hospital/Friedrich-Schiller-Universität Jena, Jena, Germany

**Keywords:** Dyspnea, Electrical impedance tomography, EIT, Long-COVID, Post-COVID

## Abstract

**Purpose and method:**

Many post-COVID patients suffer from dyspnea on exertion. To visualize exercise-induced dyspnea, a post-COVID patient and a healthy volunteer underwent an exercise test on a treadmill under stress relevant to everyday life monitored by electrical impedance tomography (EIT).

**Results:**

The lung-healthy volunteer showed an even ventilation distribution throughout the assessment, a large ventilated area, and a butterfly-like lung shape with a convex lung rim. The post-COVID patient showed clear differences in the ventilated area compared to the control subject. During exercise, a constantly changing picture of differently ventilated areas is shown. However, especially the anterior regions were under-ventilated and larger areas were partially absent from ventilation. Overall, uncoordinated breathing and an uneven distribution of ventilation dominated the findings.

**Conclusion:**

EIT is suitable for visualizing disturbed ventilation of the lungs, both at rest and under stress. The potential as a diagnostic tool in dyspnea assessment should be investigated.

**Supplementary Information:**

The online version contains supplementary material available at 10.1007/s15010-023-02062-3.

## Introduction

Dyspnea occurs in approximately 36% of post-COVID patients [1]. Guidelines recommend a pulmonary function and exercise test to clarify dyspnoeic symptoms [2]. While patients report mainly dyspnea during exertion, these tests are performed at rest [3]. Therefore, if symptoms occur under stress, an unremarkable finding at rest does not necessarily exclude a pathology under stress, especially when dynamic parameters are examined.

Electrical impedance tomography (EIT) is a radiation-free, non-invasive method to visualize lung ventilation [4]. Our working group has already shown that EIT can be used to detect pathologies in post-COVID patients [5]. However, the findings at rest can be misleading, as some pathologies only become visible on exertion. Furthermore, Scaramuzzo et al. showed that pulmonary function tests were normal in many post-COVID patients, and when comparing symptomatic and non-symptomatic post-COVID patients, no differences were found in pulmonary function parameters [6]. Therefore, we decided to develop a suitable exercise test to visualize ventilatory deficits in a post-COVID patient.

## Case

In this case report, we present a post-COVID patient suffering from dyspnea on exertion and compare the EIT findings to a healthy person. Characteristics are shown in Table 1.

**Table 1:** Characteristics of post-COVID patient and lung healthy control subject

Characteristics	Post-COVID	Healthy volunteer
Sex	Female	Female
Ethnic	White	White
Age, in years	34	38
BMI, in kg/m^2^	32.0	26.8
Months since SARS-CoV-2-infection	15	No SARS-CoV-2-infection, (not knowingly infection and seronegative)
Pre-existing conditions	Asthma bronchial (most likely infect-triggered) Thyroid operation Post-COVID-19 syndrome	Hashimoto’s thyroiditis
Medication	75 μg L-thyroxine 1–0–0 tiotropium bromide 200 2–0–0 salbutamol inhaler (if needed) 1–2 formoterol/beclomethasone 200/6 0–0–1	75 μg L-thyroxine 1–0–0

After acute COVID-19 had subsided, the patient developed a post-COVID-syndrome with dyspnea on exertion, fatigue, impaired concentration and lack of fitness. The patient had an inspiratory respiratory wheeze with a vesicular breathing sound on clinical examination. There were no findings in transthoracic echocardiogram, long-term electrocardiogram and long-term blood pressure measurement. On FACIT PROMIS Dyspnea functional limitations Short Form 10a questionnaire, the patient had 15 points. A pulmonary function test showed a slight reduction in vital capacity and a chest CT did not show pathological findings. A sit-to-stand test was performed to objectify physical performance, in which the patient was below the 2.5th percentile compared to the age/gender group.

## EIT measurement

We use the PulmoVista500 device (Fa Dräger, Lübeck/Germany). The EIT belt is placed around the thorax at the 4th intercostal space. For the EIT measurement at rest, the patient lies on a bench and breaths spontaneously for 5–10 min, while data are continuously recorded.

**Fig. 1:**
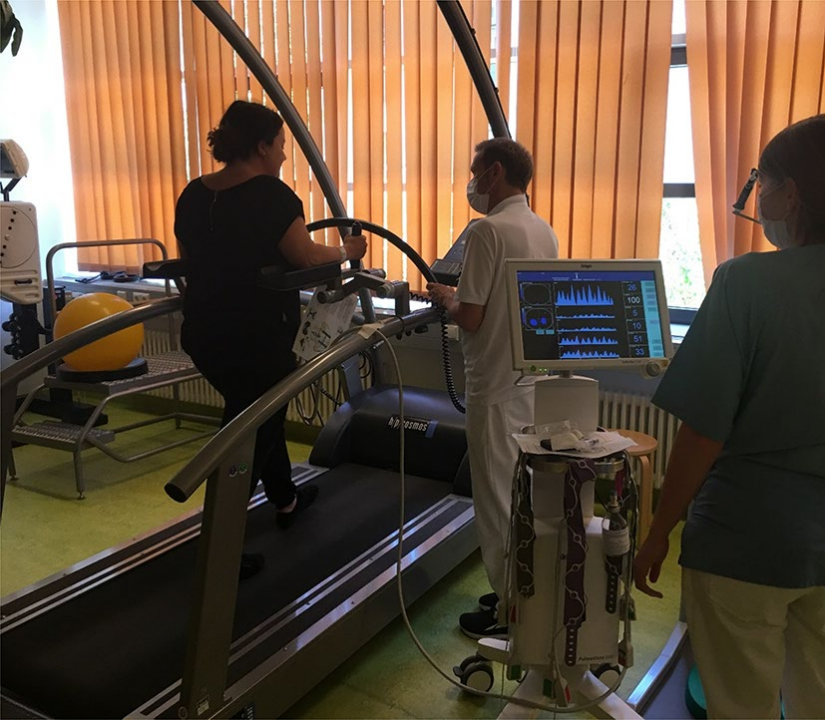
Patient wears the EIT belt at approximately the 4th intercostal space on bare skin. Before the examination, the belt with sixteen surface electrodes was wetted with 3% NaCl solution to keep the skin resistance low. To minimize artefacts, the patients’ arms were fixed. Notice: The right edge of the picture was shortened to bring the patient more into focus

**Fig. 2:**
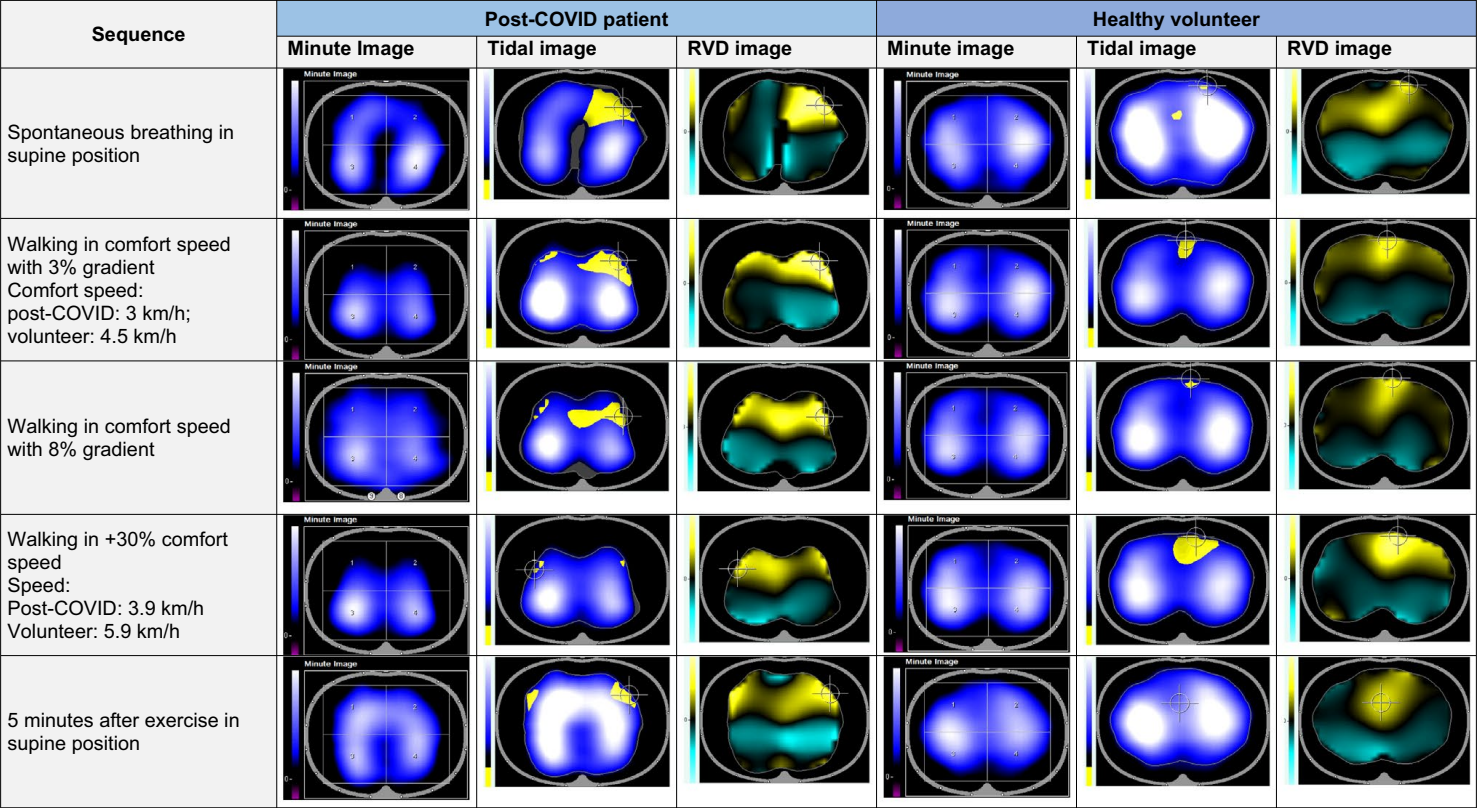
Comparing the post-COVID patient with the lung-healthy subject, there was a clear difference in the ventilated area at daily living similar exercise. The post-COVID patient had a higher regional ventilation delay (RVD), especially in the ventral left lung area, visible by the bright yellow mark. In contrast, RVD in the comparison subject is seen in a medium yellow shade in the cardiac region, presumably due to heart-related artefact. Notice: All images were created by screen recording during the evaluation using PV500 software from Fa Dräger

For the EIT measurement under exertion, the patient walks on a treadmill. To reduce movement artefacts, the forearms were put down and fixed (Fig. 1). A comfortable walking speed was determined to perform relevant to everyday life, followed by an increased intensity (Table 2; Fig. 2).

**Table 2:** Investigated parameters during EIT measurement on exertion

Measuring point		Heartbeat/minute	SpO2%	Breathing rate/minute	BORG [11]		RVD		Regional ventilation distribution^a^				Ventilation distribution	Non-ventilated areas	Pendelluft
					Stress	Dyspnea	Max	SD	ROI 1	ROI 2	ROI 3	ROI 4			
Spontaneous breathing in supine position	Patient	76	99	22			26	7	23	17	26	31	Uneven	Yes	Moderate
	Volunteer	59	99	11			15	5	23	29	23	22	Even	No	Minor
Walking at comfort speed (3 km/h) with 3% gradient	Patient	120	99	30	13	3	31	6	14	13	40	33	Uneven	Yes	Severe
	Volunteer	82	97	27	7	1	14	7	21	23	26	27	Even	No	Minor
One-minute break	Patient	99	99												
	Volunteer	54	99	16											
Walking at comfort speed with 8% gradient	Patient	130	98	29	15	5	29	8	20	16	31	26	Uneven	Yes	Severe
	Volunteer	95	97	30	12	3	14	4	22	22	27	27	Even	No	Minor
One-minute break	Patient	100	99												
	Volunteer	60	98												
Walking at + 30% comfort speed (3.9 km/h)	Patient	132	98	35	15	5	16	6	14	13	40	31	Uneven	Yes	Severe
	Volunteer	99	98	29	11	3	22	6	22	20	27	27	Even	No	Moderate
5 min after exertion, supine position	PATIENT	96	98	22			23	7	28	23	25	22	Uneven	Yes	Severe
	VOLUNTEER	50	98	14			11	5	24	28	24	21	Even	Yes	Minor

^a^Given as a percentage of approximately 100%

The following criteria were used as criteria for the evaluation: The extent of Pendelluft was categorized as minor (only one ROI affected and visible only during individual breaths), moderate (up to 2 ROIs affected and visible during every breath) and severe (3–4 ROIs affected and clearly visible during every breath). Uneven ventilation distribution was defined as 1 ROI less than or equal to 15% of tidal volume [such as the definition of He et al. [7]], or ventral–dorsal imbalance with a difference greater than 10%. Non-ventilated or under-ventilated areas are defined by visually visible lateral asymmetry of the lungs, concave retractions, or an area that has not been ventilated prior to deep inspiration.

## EIT findings

As presented in the videos, the healthy volunteer showed an even ventilation situation, no under-ventilated areas and a butterfly shaped lung with a convex lung rim. In EIT at rest, the control subject showed a small recruitable area by deeper inspiration and only slightly visible oscillating air called Pendelluft. The regional ventilation delay was side-symmetrical. All these parameters did not change during exercise.

The post-COVID patient showed an even ventilation situation at rest. Pendelluft could be detected in three of four quadrants. The patient could recruit more ventilated areas during deeper breaths than during spontaneous breathing. The maximum of regional ventilation delay was laterally asymmetric in the left ventral lung region, where distinct yellow spots could be noted in the tidal image (Fig. 2).

During exercise, ventilated areas differed constantly. However, especially in anterior regions larger areas were partially absent from ventilation. The entire lung area was now affected by oscillating air.

Five minutes after exertion, more ventilated lung area has been recruited in supine position compared to the first measurement at rest. Further parameters remained constant. The patient was exhausted after examination, agreed that this was a stress relevant to everyday life and was satisfied with the examination procedure. No adverse events occurred.

## Discussion

To our knowledge, this is the first visualization of exercise-induced dyspnea in a post-COVID patient using EIT. We demonstrate that using EIT under exercise is feasible and, therefore, is a potential tool to investigate exercise-induced dyspnea.

The question of EIT being pathologic remains unexplained. It is possible that ventilatory dysfunction may be due to neuromuscular problems. For example, the respiratory work in the post-COVID patient appears to be significantly less coordinated than that of the control. The underlying cause may be an increased anaerobic metabolism, reflected in a high post-exercise lactate and a reduced peak oxygen uptake [8, 9]. Moreover, altered autoimmune dysfunction, such as dysfunctional breathing, has been discussed in addition to deconditioning, endothelial dysfunction, tissue damage and muscular pathologies [8, 10].

Furthermore, the difficulty of imaging this respiratory distress should be noted. For example, only about 20% of post-COVID patients showed a pathology on CT or pulmonary function test at rest [3].

The main limitation of this case is that until today, there is no standardized reporting of EIT findings for spontaneous breathing. Second, the walking procedure used in our setting is not fully physiological (treadmill and fixed arms). Third, the patient has a higher BMI than the comparison subject, so that artefacts can arise from this difference. Noteworthy, as the main aim of the case report was to prove the feasibility of EIT under exertion, we decided to use this control person despite this limitation.

EIT as a radiation-free, non-invasive and cost-effective method is suitable for visualizing a disturbed ventilation of the lungs, both at rest and under stress. The potential as a diagnostic tool in assessment of dyspnea should be investigated.

### Supplementary Information

Below is the link to the electronic supplementary material.

Supplementary file1 (MP4 29486 KB) Video of the lung healthy volunteer (Video 1): In the resting measurement, the lung-healthy subject showed laterally symmetrical inspiration and expiration, an even distribution of ventilation, and a convex lung margin. Running at comfort speed, the control subject showed a physiological increase in respiratory rate and an increase in the intensely ventilated area. When walking faster and when walking with an incline, the respiratory flow appeared to be more turbulent; in part this is explained by motion-induced artifacts. At rest, a small area on the right ventral side unexpectedly appeared less ventilated after exertion

Supplementary file2 (MP4 24277 KB) Video of the post-COVID patient (Video 2): In EIT at rest, the patient showed an even ventilation situation with some special features. Oscillating air between different regions of the lung could be detected in three of four quadrants. The patient could recruit more ventilated areas in deeper breaths than in spontaneous breathing. In addition, the post-COVID patient showed a larger under-ventilated area in the left ventral region which is captured last by airflow. During exercise, a constantly changing picture of differently ventilated areas, mostly laterally symmetrical, is shown. However, especially, the anterior regions were more under-ventilated and larger areas were partially absent from ventilation. The Pendelluft phenomenon worsened because the entire lung area was now affected by oscillating air. There were clearly visible non-ventilated lung areas. Overall, uncoordinated breathing and an uneven distribution of ventilation dominated the findings

## Data Availability

Not applicable.
